# *QuickStats:* Life Expectancy at Birth, by Sex — National Vital Statistics System, United States, 2019–2021

**DOI:** 10.15585/mmwr.mm7228a5

**Published:** 2023-07-14

**Figure:**
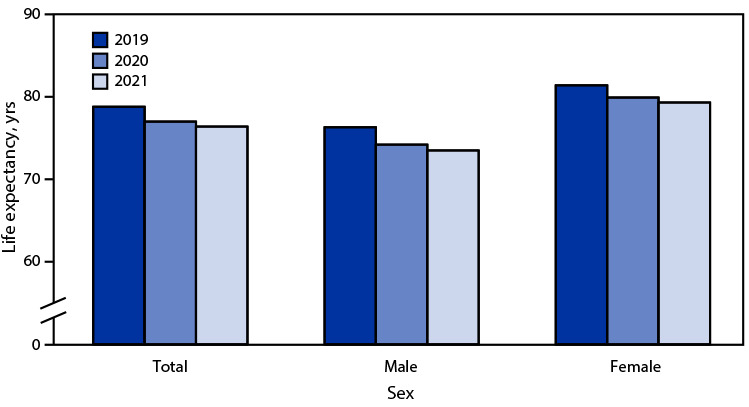
Life expectancy at birth for the total population declined 2.4 years from 78.8 in 2019 to 76.4 years in 2021. Life expectancy declined for both males and females during this period. For males, life expectancy declined from 76.3 to 73.5 years and for females from 81.4 to 79.3 years. Life expectancy was higher for females than males by 5.1 years in 2019, and that difference increased to 5.8 years in 2021.

**Sources:** National Vital Statistics System, United States Life Tables, 2020 (https://www.cdc.gov/nchs/data/nvsr/nvsr71/nvsr71-01.pdf); Mortality in the United States, 2021. https://www.cdc.gov/nchs/data/databriefs/db456.pdf

